# The temporal dynamics of humoral immunity to *Rickettsia typhi* infection in murine typhus patients

**DOI:** 10.1016/j.cmi.2019.10.022

**Published:** 2020-06

**Authors:** K. Phakhounthong, M. Mukaka, S. Dittrich, A. Tanganuchitcharnchai, N.P.J. Day, L.J. White, P.N. Newton, S.D. Blacksell

**Affiliations:** 1) Lao-Oxford-Mahosot Hospital-Oxford Tropical Medicine Research Unit, Microbiology Laboratory, Mahosot Hospital, Vientiane, Lao People’s Democratic Republic; 2) Mahidol-Oxford Tropical Medicine Research Unit, Mahidol University, Bangkok, Thailand; 3) Centre for Tropical Medicine & Global Health, Nuffield Department of Medicine, University of Oxford, UK

**Keywords:** Diagnosis, Dynamics, Humoral immunity, Immunoglobulin G, Immunoglobulin M, Lao PDR, Murine typhus, *Rickettsia typhi*

## Abstract

**Objectives:**

This study examined individuals with *Rickettsia typhi* infection in the Lao People's Democratic Republic (Lao PDR) to (a) investigate humoral immune dynamics; (b) determine the differences in reference diagnostic results and recommend appropriate cut-offs; (c) determine differences in immune response after different antibiotic treatments; and (d) determine appropriate diagnostic cut-off parameters for indirect immunofluorescence assay (IFA).

**Methods:**

Sequential serum samples from 90 non-pregnant, adults were collected at seven time-points (days 0, 7, 14, 28, 90, 180 and 365) as part of a clinical antibiotic treatment trial. Samples were tested using IFA to determine IgM and IgG antibody reciprocal end-point titres against *R. typhi* and PCR.

**Results:**

For all 90 individuals, reciprocal *R. typhi* IgM and IgG antibody titres ranged from <400 to ≥3200. The median half-life of *R. typhi* IgM was 126 days (interquartile range 36–204 days) and IgG was 177 days (interquartile range 134–355 days). Overall median patient titres for *R. typhi* IgM and IgG were significantly different (p < 0.0001) and at each temporal sample collection point (range p < 0.0001 to p 0.0411). Using Bayesian latent class model analysis, the optimal diagnostic cut-off reciprocal IFA titer on patient admission for IgM was 800 (78.6%, 95% CI 71.6%–85.2% sensitivity; 89.9%, 95% CI 62.5%–100% specificity), and for IFA IgG 1600 (77.3%; 95% CI 68.2%–87.6% sensitivity; 99%, 95% CI 95%–100% specificity).

**Conclusions:**

This study suggests suitable diagnostic cut-offs for local diagnostic laboratories and other endemic settings and highlights antibody persistence following acute infection. Further studies are required to validate and define cut-offs in other geographically diverse locations.

## Introduction

Murine typhus, caused by the obligate intracellular organism *Rickettsia typhi* is a disease transmitted to humans by the rat flea, *Xenopsylla cheopis* [1] and is a common cause of acute fever in Southeast Asia [[2], [3], [4]]. The disease has worldwide distribution but its true incidence is difficult to determine because cases are often underdiagnosed or misdiagnosed because of its non-specific clinical manifestations, usually self-limiting nature and lack of accessible diagnostic tests [1,5].

There is limited detailed literature regarding the characteristics and dynamics of humoral immunity to *R. typhi* infection, and little is known about the IgM and IgG responses in individuals with murine typhus in endemic settings. This information is important, because it can provide a better understanding of immunity and related aspects of diagnosis in the acutely ill patient.

The objectives of this study were to investigate the following topics; (a) longitudinal humoral immune dynamics following *R. typhi* infection in the murine typhus endemic setting of Lao PDR; (b) comparison of reference diagnostic results (PCR and serology) and determination of appropriate diagnostic cut-off parameters in an endemic setting for the indirect immunofluorescence assay (IFA); and (c) determination of the effect on the immune response following different antibiotic treatments in patients with *R. typhi* infection.

## Methods

### Study design and data

The data set used in this study was from a randomized clinical trial of the antibiotic treatment of murine typhus infection in Vientiane, Lao PDR [6]. An open, randomized, superiority trial was performed in adults with rapid diagnostic test evidence for infection with uncomplicated murine typhus, to compare the therapeutic efficacy of three treatment regimens: 7 days of doxycycline (Doxy7), 3 days doxycycline (Doxy3) and 3 days of azithromycin (Azith3). Non-pregnant adults (≥15 years) with positive *R. typhi* IgM rapid immunoblot tests were recruited between March 2004 and August 2009, at Mahosot Hospital, Vientiane, Lao PDR. Serum samples were aimed to be collected at approximately days 7, 14, 28, 90 180 and 365 after patient admission was completed [6].

### Ethics statement

Ethical clearance was granted by the Ethics Review Committee of the Faculty of Medical Sciences, National University of Lao PDR, Vientiane, Lao PDR and the Oxford Tropical Research Ethics Committee (OXTREC), Oxford, UK (OXTREC number 003-03).

### Laboratory assays

For the purpose of patient recruitment to the trial, an immunoblot test using the Dip-S-Ticks Murine typhus (Formerly, Cat# D-RTY03T, Panbio, Brisbane, Australia now known as ImmunoDOT *Rickettsia typhi* Cat# 800-4020, GenBio, San Diego, CA, USA) was adapted to the exclusive detection of *R. typhi* IgM using an IgG blocking agent [7], with the presence of three or four dots was considered to be IgM positive. Results were retrospectively confirmed by IFA using the *R. typhi* Wilmington strain antigen [7]. To determine quantitative *R. typhi* IgM and IgG end-points in the longitudinal serum collections, samples were titrated in the IFA from <400, 400, 800, 1600, ≥3200 and the highest dilution at which specific fluorescence could be observed was considered the end-point [6]. To demonstrate the *R. typhi* organism, EDTA buffy coat samples underwent Genomic DNA extraction using the DNeasy Blood & Tissue Kit (Qiagen, Qiagen Str. 1, 40724 Hilden, Germany) followed by detection of the 17-kDa gene of *Rickettsia* spp. [8].

### Data analysis

Data were analysed using R software (version 3.3.0) [9]. The 95% CI for the median reciprocal titres of *R. typhi* IgM and IgG were calculated and superimposed on the immune response plots to compare the overall immune response characteristics. Bayesian latent class models were used to determine sensitivity and specificity of different diagnostic cut-offs and to select the optimal cut-off titres. The antibody half-life (*t*_½_) was calculated for each patient and, with the exception of those that demonstrated no change in antibody titres, using the pharmacokinetic data command *rpkexamine* and the summary statistics command *summary, detail* to determine median and interquartile ranges (IQR), respectively, using STATA 15.1 (Statacorp, College Station, TX, USA). Statistically significant (p < 0.05) differences in the reciprocal titre diagnostics, treatment and antibody isotype groups were determined using the Wilcoxon Rank Sum test.

## Results

### Patient and sample characteristics

**Fig. 1:**
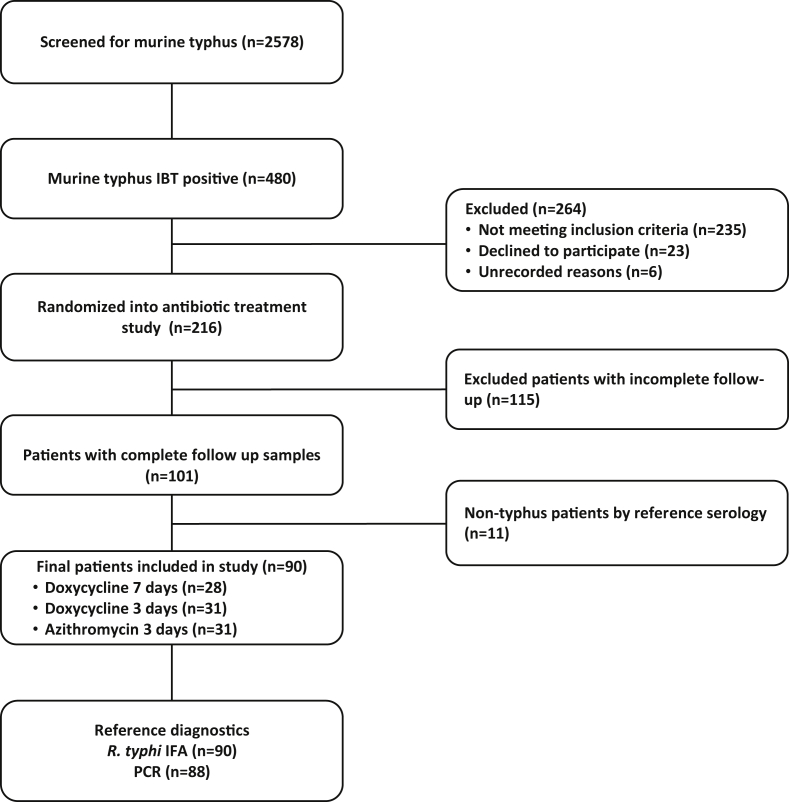
The flowchart of the study including clinical trial details.

A total of 489 individuals were immunoblot test positive, of whom 264 (55%) did not meet the inclusion criteria of the trial (Fig. 1) for various reasons [6]. Samples from 216 individuals were eligible and of these, 101 (52.9%) had samples collected at all time-points and were therefore eligible for inclusion in this study. Of the 101 immunoblot test positive individuals, 11 (11%) were considered to be not murine typhus patients following reference diagnostics. Therefore, a final cohort of 90 murine typhus patients were included in this study and serum samples were collected at all requisite time-points. Eighty-eight (98%) of the 90 patients were tested by PCR on admission and of these 29 (29/88; 33%) were PCR-positive (PCR + ve). Two patients (2%) were not tested by PCR techniques because of a lack of available technology and appropriate samples at the start of the study. The median days of illness and IQR before presentation was 8 days (IQR 7–10 days). The collection of longitudinal post-admission samples (i.e. approximately days 7, 14, 28, 90 180 and 365) at each time-point was made on days 6 (IQR 4–7), 14 (IQR 14–15), 28 (IQR 28–30), 91 (IQR 90–96), 183 (IQR 181–189) and 368 (IQR 365–378), respectively.

### Immune dynamics—overall

**Fig. 2:**
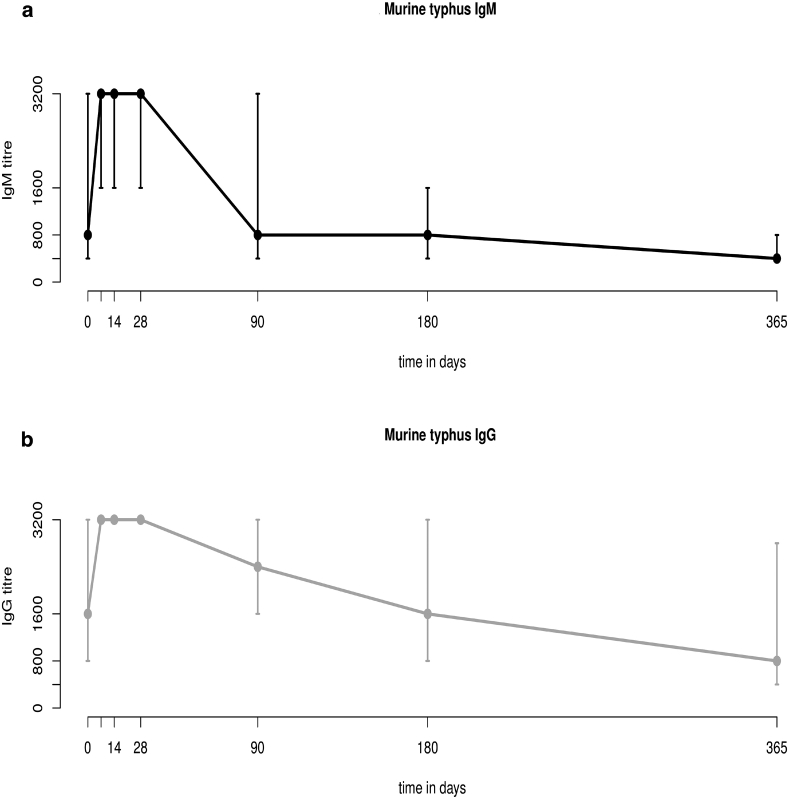
Behaviour of IgM and IgG immune responses in patients with murine typhus by day of follow up. Mean reciprocal titres are displayed in black and grey with error bars displaying maximum and minimum values. Black lines indicate IgM and grey lines represent IgG. The *x*-axis represents time in days, and the *y*-axis represents IgM and IgG reciprocal titres, ranging from 399 to 3200.

**Fig. 3:**
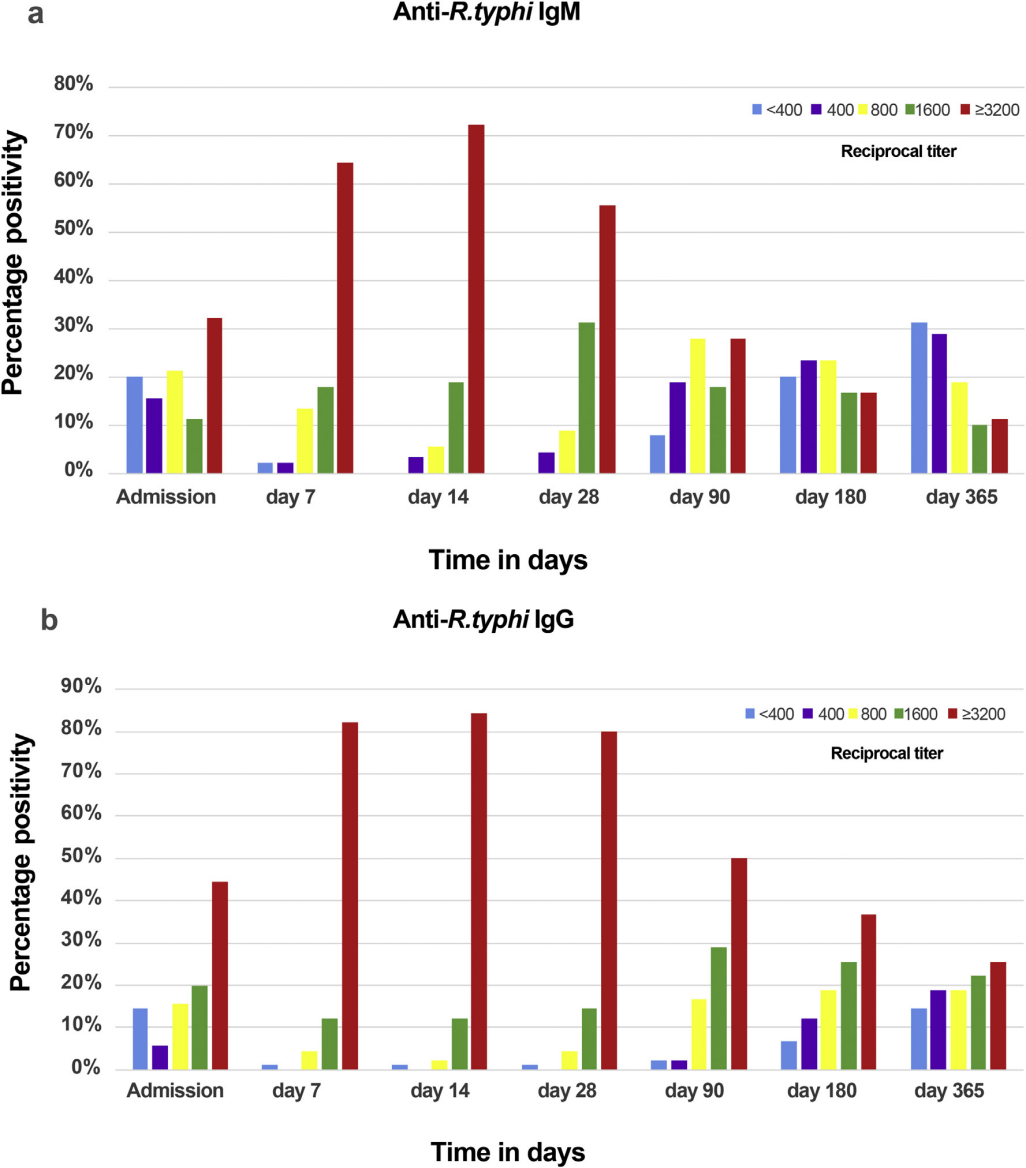
The distributions of IFA reciprocal titres in patients with murine typhus at each follow-up time-point. The *y*-axis represents frequency in percentage and the *x*-axis represents time in days. Different colours indicate each reciprocal titer as follows <400 (blue), <400 (purple), 800 (yellow), 1600 (green), ≥3200 (red).

Reciprocal *R. typhi* IgM and IgG antibody titres for the longitudinal post-admission sample collections ranged from <400 to ≥3200 (Table 1, Fig. 2a,b). The median *t*_½_ of *R. typhi* IgM was 126 days (IQR 36–204 days) and of IgG was 177 days (IQR 134–355 days). Twenty-two patients demonstrated no change in antibody titres and were excluded from the median half-life calculation Overall median patient titres for *R. typhi* IgM (Fig. 2a) and IgG (Fig. 2b) were significantly different (p < 0.0001) and at each temporal sample collection point (p < 0.0001 to p 0.0411) (Table 1). IgM and IgG titres demonstrated generally lower antibodies titres on admission, with increasing titres within the 2 weeks following discharge (Fig. 3a,b). On admission, 32.2% (29/90) of patients had a maximum anti-*R. typhi* IgM titre ≥3200, followed by 64.4% (58/90), 72.2% (65/90) and 55.5% (50/90) on days 7, 14 and 28, respectively, declining thereafter from day 90 to day 365 (Fig. 3a). For IgG, 44.4% (40/90) of patients demonstrated a maximum IFA admission titre, that increased to 82.2% (74/90), 84.4% (76/90) and 80% (72/90) on days 7, 14 and 28, respectively, and declined until day 365 (Fig. 3b).

**Table 1:** Median *Rickettsia typhi* reciprocal IgM and IgG titres for patients with murine typhus at the follow-up sample collection time-points

Follow-up time-point (days)	Median days (IQR)	*Rickettsia typhi* median reciprocal titres (95% CI)		Wilcoxon rank-sum (p)
		IgM	IgG	
0	0	800 (800–1600)	1600 (1600–3200)	0.0173
7	6 (IQR 4–7)	≥3200 (3200–3200)	≥3200 (3200–3200)	0.0046
14	14 (IQR 14–15)	≥3200 (3200–3200)	≥3200 (3200–3200)	0.0411
28	28 (IQR 28–30)	≥3200 (1600–3200)	≥3200 (3200–3200)	0.0005
90	91 (IQR 90–96)	800 (800–1600)	2400 (1600–3200)	<0.0001
180	183 (IQR 181–189)	800 (<400–800)	1600 (1600–1600)	<0.0001
365	368 (IQR 365–378)	<400 (<400–800)	800 (800–1600)	0.0001

Abbreviation: IQR, interquartile range.

### Effect of reference diagnostic result

**Fig. 4:**
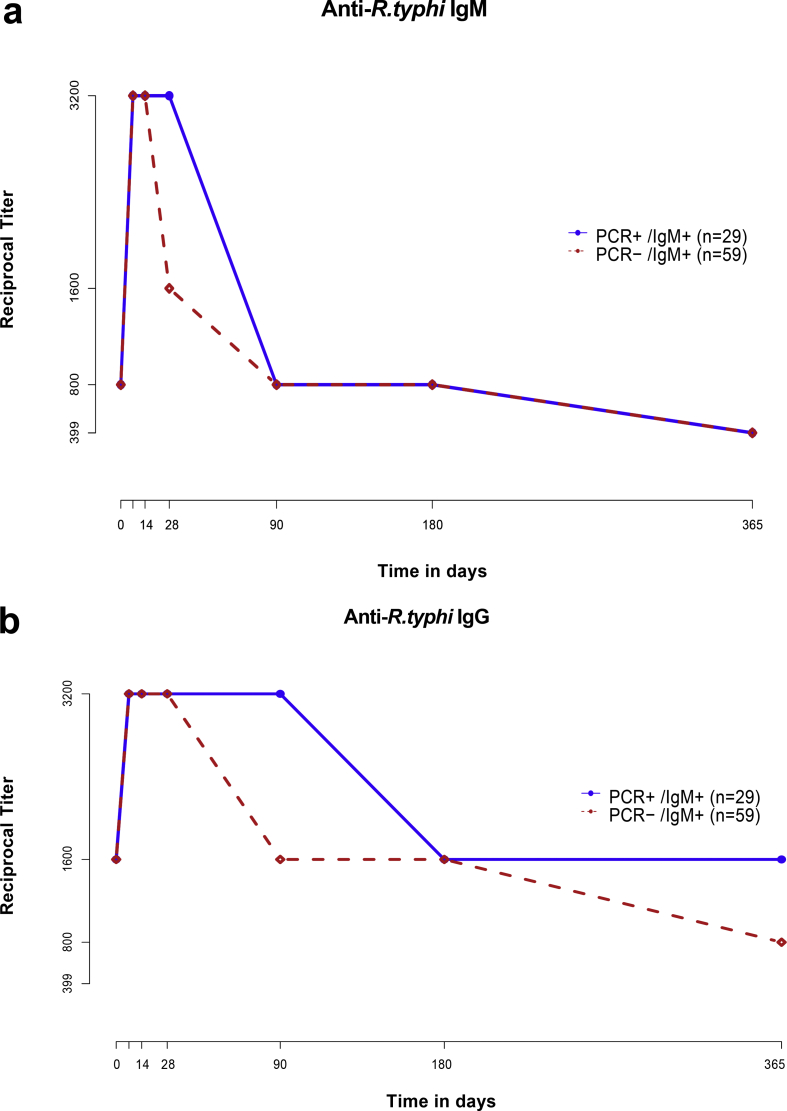
Comparison of the median immune response for anti-*Rickettsia typhi* IgM (a) and IgG (b) over time comparing PCR-negative and PCR-positive patients.

Generally, median antibody responses for PCR + ve patients appeared to be more sustained for both *R. typhi* IgM and IgG antibodies when compared with PCR-negative (PCR–ve) patients (Fig. 4a,b). IgM titres in PCR + ve patients (median *t*_½_ = 123 days, IQR 34–163 days) were not significantly different (p 0.168) from PCR–ve patients (median *t*_½_ = 163 days, IQR 40–208 days). However, IgG titres in PCR + ve patients were significantly different (p 0.030) when compared with PCR–ve patients. (*t*_½_ CR + ve 125 days, IQR 125–404 days versus *t*_½_ PCR–ve 193 days, IQR 54–308 days).

### Effect of antibiotic treatments

**Fig. 5:**
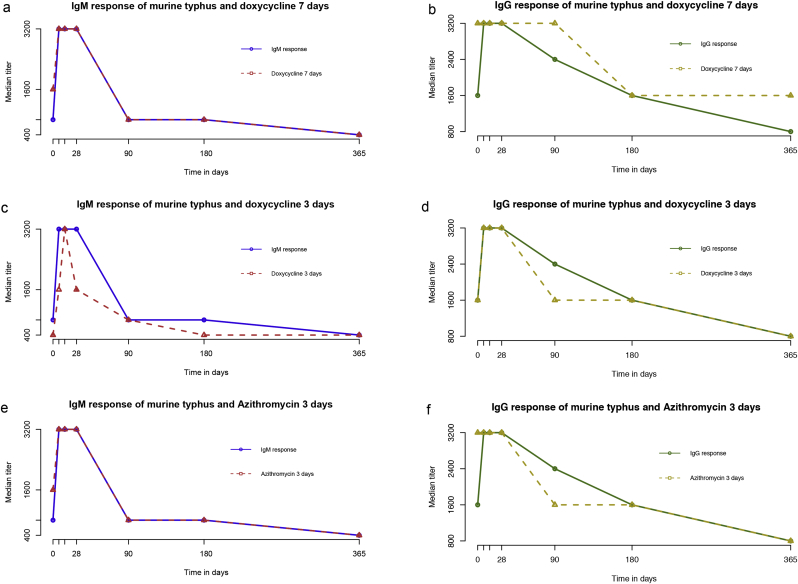
Comparison of the median immune response for anti-*Rickettsia typhi* and three different treatment regimens.

Immune response for both *R. typhi* IgM and IgG antibodies in murine typhus patients for three different antibiotic treatment regimens are presented in Fig. 5. Results of the original study demonstrated that azithromycin was inferior to doxycycline as oral therapy for uncomplicated murine typhus and Doxy3 and Doxy7 regimens had similar efficacies [6]. Of the 90 patients, 28 (31.1%) were treated with Doxy7, 31 (34.4%) with Doxy3 and 31 (34.4%) with Azith3.

The IgM immune responses to *R. typhi* for the three treatment groups were significantly different (p 0.0001). Median IgM immune responses for Doxy7 (Fig. 5a) and Azith3 (Fig. 5e) demonstrated similar patterns with median *t*_½_ of 134 days (IQR 35–288 days) and 125 days (IQR 44–188 days), respectively. Maximum median titres rapidly reached ≥3200 following admission and remained constant for approximately 28 days with antibody decay identical to the overall IgM trend. This contrasted with patients who were treated with Doxy3 (Fig. 5c), where anti-*R. typhi* IgM peaked at day 14 with a more pronounced antibody decay (median *t*_½_ = 89 days, IQR 35–218 days). IgG immune responses to *R. typhi* for the three treatment groups were also significantly different (p 0.0001). Doxy7 IgG (Fig. 5b) had a maximum median titre until day 90 (median *t*_½_ = 158 days, IQR 114–280 days). Doxy3 and Azith3 patients similar had IgG dynamic responses (Fig. 5d,f) with pronounced reductions in the duration of maximum median titres compared with overall results (median *t*_½_ 76 days, IQR 102–312 days and 202 days, IQR 119–414, respectively).

### Estimation of diagnostic cut-offs

The optimal admission sample diagnostic cut-offs were determined using Bayesian latent class models (Table 2). An admission IFA IgM of ≥3200 had the lowest sensitivity of 41.4% (95% CI 35.8%–50.8%) followed by 55.7% (95% CI 48.1%–67.2%), 78.6% (95% CI 71.6%–85.2%) and 88.9% (95% CI 85.4%–91.1%) for IgM of 1600, 800 and <400, respectively. Optimal specificity of 99% (95% CI 100%–100%) was in the range of ≥3200 and 1600 that decreased to 89.9% (95% CI 62.5%–100%) at 800. Similar to IgM, an IgG admission IFA of ≥3200 had the lowest sensitivity of 53.4% (95% CI 47%–60.6%) followed by 77.3% (95% CI 68.2%–87.6%), 87.5% (95% CI 84.7%–90.1%) and 91.4% (95% CI 88.8 %–92.7%) for IgG of 1600, 800 and <400, respectively. Both IFA IgG of ≥3200 and 1600 had the highest specificity of 99% (95% CI 95%–100%), followed by 70.5% (95% CI 38.8%–100%) and 58.6% (95% CI 26%–100%) for the IgG of 800 and <400, respectively. Balancing the requirements for optimal sensitivity and specificity, we recommend a diagnostic cut-off of 800 for IgM and 1600 for IgG.

**Table 2:** The sensitivity and specificity of a range of diagnostic cut-off reciprocal titres of IFA IgM and IgG using Bayesian latent class models for the true diagnosis of murine typhus in admission samples

Reciprocal IFA titre	IgM cut-off		IgG cut-off	
	Sensitivity (%) (95% CI)	Specificity (%) (95% CI)	Sensitivity (%) (95% CI)	Specificity (%) (95% CI)
≥3200	41.4 (35.8–50.8)	99 (100–100)	53.4 (47–60.6)	99 (100–100)
1600	55.7 (48.1–67.2)	99 (96.9–100)	77.3 (68.2–87.6)	99.5 (95–100)
800	78.6 (71.6–85.2)	89.9 (62.5–100)	87.5 (84.7–90.1)	70.5 (38.8–100)
<400	88.9 (85.4–91.1)	59.2 (31.2–100)	91.4 (88.8–92.7)	58.6 (26–100)

Abbreviation: IFA, immunofluorescence assay.

## Discussion

This study characterized the longitudinal dynamics of human *R. typhi* IgM and IgG immune responses among murine typhus patients in Vientiane, Lao PDR. The median *t*_½_ of *R. typhi* IgM was 126 days (IQR 36–204 days) and IgG was 177 days (IQR 134–355 days) and based on these results we calculated diagnostic IFA cut-off reciprocal titres of anti-*R.typhi* IgM 800 and IgG 1600 for admission sera in the murine typhus endemic setting of Vientiane, Lao PDR.

Before this study, there was very limited knowledge of the *R. typhi* longitudinal humoral immune dynamics in humans, reports having been on individual cases or small sample sizes. The most complete *R. typhi* longitudinal humoral immunity data before our study was that of a New Zealand murine typhus patient with IgM and IgG reciprocal titres of 80 and 1280 on admission, titres of 2560 and 1280 at day 26, and titres of 40 and negative at 13 months, respectively [10]. In a similar US report [11], a murine typhus patient had a reciprocal IFA titre of 4096 on admission, and 4 months later the titre had decreased eight-fold to 512. In a study for the development of a new *R. typhi* ELISA, Halle and Dasch [12] demonstrated consistent IgM and IgG titres in samples from two patients collected 1 year apart when tested by ELISA, microagglutination test and complement fixation using various antigen preparations. A more detailed study by Nelson [13] that was specifically designed to characterize the early immune response (days 0–22) in individuals with non-fatal murine typhus in the southeastern USA (*n* = 2) reported similar observations (maximum positivity at 12–16 days; exact titres not reported) although the features of antibody decay dynamics were not described.

The IFA has become the default reference standard serological assay for murine typhus since its description in 1959 [14]; however, there has been minimal investigation or discussion justifying the use of diagnostic cut-offs. Here we have determined the IFA cut-off reciprocal titre to provide an evidence-base for the diagnosis of murine typhus in the endemic setting of Vientiane, Lao PDR [15]. Using a Bayesian latent class model approach, we selected the optimal IFA cut-off reciprocal titre of 800 for IgM with a resultant sensitivity of 78.6% and a specificity of 89.9%. For IgG, the sensitivity and specificity using an IFA cut-off reciprocal titre of 1600 were 77.3% and 99.5%, respectively. Murine typhus studies in Asia have inconsistently applied diagnostic cut-offs for when using the IFA (see Supplementary material, Table S1). IFA cut-off reciprocal titres from previous studies for IgM ranged from ≥64 [16] to 400 [7] and for IgG ranged from ≥128 [16] to ≥400 [3]. Whole antibody (IgM and IgG) was consistently applied at ≥400 in reported studies [[17], [18], [19]]. In Lao PDR, previous studies have used cut-offs of ≥64 or ≥400 for IgM and ≥128 for IgG with or without a ≥4-fold dynamic rise between sample collections [16]. A dynamic rise in titre between paired samples was also regarded as indicative of an active infection. However, some studies indicated a minimum titre (up to 200 in the latter sample) or IgG only (see Supplementary material, Table S1). In areas where murine typhus is endemic, the issue of residual antibodies from previous infections causes difficulty when performing diagnosis of acute disease using serological methods. The prevalence of anti-*R. typhi* IgG antibodies in Vientiane was 20.6% in healthy individuals [15], demonstrating an issue with residual antibodies. However, selection of cut-off titres remains controversial and tends to be dependent on the seroprevalence in the population (see Supplementary material, Table S1) [20].

Comparison of anti-*R. typhi* IgM and IgG immune responses indicated that *R. typhi* PCR + ve patients generally had a prolonged immunity compared with PCR–ve patients. This may be a result of the increased rickettsial load in PCR + ve patients that, in turn, provided greater immunogenic stimulus resulting in an increased immune response. In this study, only 33% of the patients were PCR + ve. Given the relatively low sensitivity, other studies have demonstrated the value of performing PCR and serology for admission samples [21,22].

This study also examined the immune response following three different antibiotic treatments. Significant differences in the IgM and IgG immune responses following antibiotic treatments were noted, but there was no pattern related to either days of treatment (Doxy3 versus Doxy7) or antibiotic (Doxy versus Azith). Both doxycycline [[23], [24], [25]] and azithromycin [26] are known to modulate the immune response and are both bacteriostatic in action. It is therefore unclear what is the true effect of these treatments that was compounded by the lack of a placebo arm to contrast the immune response of untreated patients.

Strengths of the study include the complete longitudinal sample collections over a period of 1 year, which is the longest of any such a study, and both PCR and IFA diagnostics were performed for acute patient samples. However, there are limitations associated with our study. This study focused on inpatients at one hospital in Vientiane, Lao PDR and only approximately half of the trial patients were included in the analysis after exclusions. Only adult inpatients were recruited and our data did not determine the level of background immunity to murine typhus in healthy Lao populations. Due to resource limitations, we only tested a restricted range of serum dilutions (i.e. 400, 800, 1600, 3200) rather than determining an end-point titre for all samples. Another limitation is the wide confidence intervals and IQR for the median reciprocal titre that probably contributed to the disparity between immune response plots and antibody half-life results, especially in the comparison of the immune response of the antibiotic treatment groups.

Results presented from this study will inform diagnostic laboratories in Lao PDR and other settings where murine typhus is endemic regarding locally appropriate diagnostic cut-offs and will underline the diagnostic problems with antibody persistence following acute infection. It is expected that the diagnostic cut-offs calculated here will be adopted nationally in Lao PDR. Further prospective studies are needed to validate these and to define cut-offs in other geographically diverse locations. Until the studies have been performed, any diagnostic interpretations should be made with caution, especially for travellers from non-endemic regions.

## Transparency declaration

There are no personal conflicts of interest.

## Funding

All authors are funded by the 10.13039/100010269Wellcome Trust of the UK, Grant number 106698/Z/14/Z.
