# Supplementary material for: The temporal dynamics of humoral immunity to Rickettsia typhi infection in murine typhus patients
Source: Clin Microbiol Infect. 2020 Jun;26(6):781.e9–781.e16. doi: 10.1016/j.cmi.2019.10.022 (PMC7284305; doi:10.1016/j.cmi.2019.10.022)
Supplement: Multimedia component 1 [file mmc1.doc]

Table S1. Immunofluorescence assay diagnostic criteria applied for the diagnosis of murine typhus infection in Asia.

| Location | Year | Prevalence (%) | Diagnostic criteria | | | | Study |
| --- | --- | --- | --- | --- | --- | --- | --- |
|  |  |  | IgM | IgG | Whole Ab | Dynamic rise |  |
| Thailand | 1990 | 79.3 |  |  | ≥400 | ≥4-fold to minimum of ≥200 | (17) |
| Thailand | 1993 | 100* |  |  | ≥400 | ≥4-fold to minimum of ≥200 | (18) |
| Thailand | 1995 | 61.1 |  |  | ≥400 | ≥4-fold to minimum of ≥200 | (19) |
| Laos | 2006 | 9.6 | ≥64 | ≥128 |  | ≥4-fold | (16) |
| Taiwan | 2008 | 0 | ≥80 |  |  | ≥4-fold IgG | (27) |
| Indonesia | 2009 | 6.6 | ≥64 | ≥256 |  | ≥4-fold | (28) |
| Laos | 2010 | 17.8/32.9** | ≥400 |  |  | ≥4-fold | (20) |
| Taiwan | 2012 | 100* | ≥80 |  |  | ≥4-fold IgG | (29) |
| Vietnam | 2015 | 33.3 |  | ≥400 |  | ≥4-fold IgG | (3) |
| Taiwan | 2017 | 20.0 | ≥80 |  |  | ≥4-fold IgG | (30) |

References

[27] Lai CH, Huang CK, Weng HC, Chung HC, Liang SH, Lin JN, et al. Clinical characteristics of acute Q fever, scrub typhus, and murine typhus with delayed defervescence despite doxycycline treatment. Am J Trop Med Hyg 2008;79:441–446.

[28] Gasem MH, Wagenaar JF, Goris MG, Adi MS, Isbandrio BB, Hartskeerl RA, et al. Murine typhus and leptospirosis as causes of acute undifferentiated fever, Indonesia. Emerg Infect Dis 2009;15:975–977.

[29] Chang K, Chen YH, Lee NY, Lee HC, Lin CY, Tsai JJ, et al. Murine typhus in southern Taiwan during 1992–2009. Am J Trop Med Hyg 2012;87:141–147.

[30] Chang K, Lee NY, Ko WC, Lin WR, Chen YH, Tsai JJ, et al. Characteristics of scrub typhus, murine typhus, and Q fever among elderly patients: prolonged prothrombin time as a predictor for severity. J Microbiol Immunol Infect 2019;52:54–61.
